# Adherence to Psychological First Aid after Exposure to a Traumatic Event at Work among EMS Workers: A Qualitative Study

**DOI:** 10.3390/ijerph182111026

**Published:** 2021-10-20

**Authors:** Marine Tessier, Josianne Lamothe, Steve Geoffrion

**Affiliations:** 1 Department of Psychology, Université de Montréal, Montréal, QC H3T 1J4, Canada; 2 Trauma Studies Center, University Institute of Mental Health of Montreal, Montréal, QC H1L 2K4, Canada; josianne.lamothe@mail.mcgill.ca (J.L.); s.geoffrion@umontreal.ca (S.G.); 3 School of Criminology, Universiteé de Montréal, Montréal, QC H3T 1J4, Canada; 4 School of Psychoeducation, Université de Montréal, Montréal, QC H3T 1J4, Canada

**Keywords:** emergency medical services workers, early post-trauma intervention, first responders, psychological first aid, peer support, implementation, adherence, sustainability

## Abstract

Managing post-traumatic stress reactions in the first few days after exposure to a potentially traumatic event in the course of one’s work remains a challenge for first responder organizations such as Emergency Medical Services (EMS). Psychological First Aid (PFA) is an evidence-informed approach to reducing initial distress and promoting short- and long-term coping strategies among staff in the aftermath of exposure. PFA provided by peer helpers is considered a promising solution for first responder organizations. Unfortunately, first responders may encounter stigma and barriers to mental health care. Therefore, a deeper investigation is needed regarding adherence over time to implemented PFA intervention. The purpose of this study is to qualitatively explore factors that influence adherence to PFA intervention of recipients and peer helpers. EMS workers (n = 11), working as PFA peer helpers for one year, participated in semi-structured interviews. Data were analyzed using thematic analysis; intercoder reliability (κ = 0.91) was also used. Researchers identified four themes and 11 subthemes influencing adherence to PFA intervention: (1) individual perceptions and attitudes of peer helpers and recipients about pfa intervention; (2) perceived impacts on peer helpers and recipients; (3) organizational support to pfa intervention; and (4) congruence with the occupational culture. Study findings herein suggest that it is conceivable to act on various factors to improve adherence to PFA intervention among peer helpers and recipients within EMS organization. This could lead to enhanced understanding of the challenges involved in sustaining a peer led PFA program for first responders.

## 1. Introduction

First responders such as Emergency Medical Service (EMS) workers respond to a broad range of emergencies as part of their mandate. These emergencies are unpredictable and recurrent in their nature, increasing the risk of exposure to highly disturbing events (i.e., seeing someone die, a badly beaten adult, or completing a death notification; [1]), and even more so during a prolonged crisis (i.e., disease pandemic; [2]). Mental health problems resulting from exposure to a traumatic event may include acute stress disorder, post-traumatic stress disorder (PTSD), anxiety, depression, and substance abuse [1,3]. Among first responders (i.e., police officers, EMS workers, and firefighters), EMS workers appear worldwide to be the population most at risk of developing PTSD [3]. A meta-analysis estimated prevalence rates of 11% for PTSD, 15% for depression, 15% for anxiety, and 27% for general psychological distress among EMS workers [4]. In addition to mental health problems, meta-analyses have highlighted the negative health impacts of PTSD on physical well-being in adults [5] and among first responders specifically [6,7]. Repercussions are also felt at the organizational level (e.g., increased sick leaves, lowered operational performance; [8,9]). Despite these alarming consequences, a gold standard for early organizational intervention among exposed EMS workers has not yet been established [4,10].

One of the challenges in implementing early post-trauma interventions in organizations might be the mental health-related stigma and the barriers to mental health care experienced by a significant proportion of first responders, including EMS workers [11], which interfere with them getting help even when help is readily available. Several studies have shown how organizational support might be a protective factor against mental health symptoms after exposure to workplace trauma [12]. Likewise, EMS workers receiving organizational support report greater emotional stability and belongingness, a steeper decrease of post-traumatic symptoms, and a great likelihood of achieving post-traumatic growth [13,14]. International guidelines also recognize the protective role of peer support following a traumatic event in first responder organizations [15,16].

If peer support appears to be a promising solution to operationalizing social support within EMS organizations, the management of post-traumatic stress reactions in the first days following a traumatic event remains difficult. Organizations are confronted with a lack of effective early post-traumatic intervention options to suit and cover all individuals exposed to a traumatic event, regardless of symptomatology, as recently stated by a meta-analysis [10]. Since the 1990s, psychological debriefing has been proposed to address adverse reactions in the aftermath of a traumatic event [17]. This type of intervention is still widely used in first responder organizations [18]. However, considerable controversy exists regarding the potential effects of this form of intervention [18,19]. Instead, multiple health organizations and international trauma experts recommend providing Psychological First Aid (PFA) after a traumatic event since it is an evidence-informed early intervention approach [20,21].

Initially developed as a response to natural disasters and terrorist attacks, PFA aims to reduce the initial distress caused by traumatic events and foster short- and long-term adaptive coping strategies. It was designed to help victims of traumatic events and first responders on-site and can be delivered with minimal training in mental health intervention [20]. However, a systematic review concluded that further investigation was needed to establish the effectiveness of PFA on mental health [22]. Indeed, relatively few studies have evaluated this type of intervention, especially regarding the potential reduction in post-traumatic stress symptoms [23,24,25,26]. On this note, it is important to remember that PFA is not a form of treatment, but an early intervention; therefore, more than symptom reduction, PFA aims to foster better adaptation in the short and long term. Therefore, it remains relevant to investigate its implementation in high-risk organizations before examining its effectiveness on multiple outcomes.

Researchers and practitioners view peer-led PFA as a promising solution in first responder organizations, but empirical evidence as to its implementation in high-risk organizations is still in its infancy [27,28]. Forbes and colleagues [27] developed a framework to implement PFA within high-risk organizations, but no studies have yet demonstrated the ecological validity of their framework. The few studies evaluating PFA generally investigate the effectiveness of PFA training on providers’ confidence and the skills required to respond after a traumatic event appropriately [28,29,30,31]. However, no study has evaluated PFA implementation, denying researchers important information on the potential facilitators and barriers to implementing and ensuring PFA adherence over time (i.e., peer facilitators’ and service users’ active engagement with PFA support).

As first responder organizations increasingly move toward the adoption of evidence-informed mental health interventions, researchers should study how they are implemented and adhered to by EMS workers. On this note, a recent scoping review identified three key components using meta-ethnography analysis [12]. Richins and colleagues [12] found that respect for the organizational culture, support from the host organizations, and building on existing social cohesion and peer support systems made implementation easier, in emergency response organizations. Although this review did not include any PFA intervention, those recommendations for delivering and evaluating early post-trauma interventions may be helpful to put into perspective with our investigation about factors that influence a key part of sound implementation, namely adherence. Adherence is a crucial concept in health practice [32,33] as interventions evolve positively or negatively according to the context in which they occur. Investigating implementation without considering adherence over time regarding participation appears to be in vain [34]. Even if there is still little agreement on a conceptual definition of adherence in the health disciplines [32], it is generally referred to as “the extent to which patients follow the instructions they are given for prescribed treatments” [35] (p. 2). In the present study, we use the concept of adherence in a slightly larger perspective, as the extent to which EMS recipients as well as peer helpers both follow the organizational recommendations to be involved in PFA intervention after exposure to a traumatic event. In order for PFA programs to be sustainable and to become routine inside EMS organizations, it is important that those who benefit from and those who volunteer to provide PFA intervention maintain their involvement over time. As we previously stated, there is still little scientific knowledge about PFA intervention in organizational setting. No studies have yet demonstrated the ecological relevance or transferability of the suggested frameworks. Such a problematic can be better approached in its complexity by a qualitative research design. Qualitative approaches are particularly valuable when you want to explore a new field of knowledge and remain as close as possible to the participants’ experience [36]. Therefore, the present study aims to qualitatively identify factors that influence the adherence of peer helpers and recipients to PFA intervention in an EMS organization by exploring peer helpers’ perspectives.

## 2. Materials and Methods

### 2.1. Participants

Our 11 participants were recruited from a pool of 37 PFA trained peer helpers of one EMS organization [Urgences-santé], one of Canada’s largest emergency medical services. These peer helpers were active EMS workers trained, using Brymer’s PFA manual [20], to provide PFA to their colleagues after exposure to a traumatic event in the course of their work, doing so in the early hours after the event (i.e., on site, or back at the station). From June 2018 to September 2018, the organization’s in-house psychologist conducted a 14-h PFA training in group sessions (8–10 participants). The research interviews took place one year after implementation of PFA. Considering our small population, we have tried to ensure internal diversification using opportunistic sampling (according to sex, age, type of job, and professional experience) [36,37]. To capture potential differences in perspective due to the changing culture of mental health care among first responders, we ensured that the number of years of experience was diversified among our participants. Furthermore, because PFA was a new intervention in the organization, few PFA interventions had occurred each month since the peer helpers were trained, so we needed to ensure that our participants had provided a minimum of 3 PFA interventions before recruiting them to ensure their ecological experience as PFA peer helpers. All participants reported that they had provided between three and 20 PFA interventions between August 2018 and September 2019. The researchers did not seek to obtain a statistically representative view of the study population, but rather a portrait representative of the diversity of possible experiences.

### 2.2. Procedure 

The interviews took place in August and September 2019. All PFA-trained peer helpers were informed about the study by the organization’s psychologist. The researchers did not have access to the reasons for potential participants’ refusals at this stage. Subsequently, the researchers sent more information about the study through emails and phone calls to those who voluntarily gave preliminary agreement. The project had ethical approval (CER-CEMTL 2019-1884) from the ethics committee of Integrated University Health and Social Services Centre for the East Island of Montreal. Participants were informed of the process and intentions of the study, signed a consent form, and were each given a personal code for anonymity. No participant refused or dropped out of the study at this stage or afterwards. Interviews were conducted over the phone at only one point in time per participant. They were asked to be in a quiet and confidential setting outside of their working hours. Telephone interviews were selected as the data collecting method for practical reasons, especially since it facilitates scheduling meetings for the workers and the research team. Moreover, current evidence shows that telephone interviews do not produce lower quality data than face-to-face interviews [38]. We stopped recruiting after a satisfactory level of information power was reached. Information power dictates that the more information the sample holds, the lower number of participants is needed, based on five criteria (i.e., aim of the study, sample specificity, established theory, quality of dialogue, analysis strategy) [39]. Without being able to claim the data saturation, the authors considered that the collected data allowed to add substantial information to the studied phenomenon.

### 2.3. Materials

The first author conducted individual semi-structured interviews lasting between 30 and 45 min each. All interviews were audio-recorded and later transcribed for qualitative analysis purposes. The interview grid was developed based on PFA intervention implementation, practicability, acceptability, and the consequences of PFA intervention. The research question was inductively developed from the participants’ answers to these questions. The interviewer used reflection and reformulation strategies to explore further following the interview schedule flexibly. Participants also filled out a short sociodemographic questionnaire.

### 2.4. Data Analysis

Researchers used an inductive thematic analysis approach to analyze the interview data. Thematic analysis is especially useful in understudied and descriptive qualitative research. It allows for the identification, analysis, and the report of patterns within data while being independent from theoretical frameworks. This flexibility allows a more accessible sharing and understanding of data—process and results—with people from all research backgrounds (i.e., researchers of different theoretical orientation, stakeholders, knowledge users) [40]. Inductive thematic analysis was favored by the authors because it is data-driven, meaning that it allows researchers to move away from the pre-existing coding framework and possible preconceptions [40]. The six phases of thematic analysis were followed: familiarization with the data, generation of preliminary codes, searching for potential themes, reviewing themes, defining and naming themes, and producing the report [40]. Only one interviewer, information power, and sample diversity were employed as strategies to ensure the reliability and transferability of the results [36,41]. Moreover, all transcripts were coded systematically by the first author. The last author reviewed the suggested themes, and disagreements led to reexamining the data until the raters reached an agreement. Subsequently, the second author performed double coding. Cohen’s kappa was chosen to measure the intercoder reliability of themes as it is a strict measure [42]. Eleven suggested subthemes were coded using 27% of the interview materiel (i.e., three transcripts), all randomly selected [43]. The global Cohen’s Kappa was κ = 0.91 for inter-rater correlation (SD = 0.0925). Kappa values between 0.40 and 0.60 are commonly considered satisfactory agreement, and values above 0.80 suggest perfect agreement [44]. Our intercoder reliability is therefore considered sufficient to continue data interpretation. Thematic analysis was performed using QDA Miner 5.0 software package (Provalis Research, Montreal, QC, Canada) and intercoder reliability was supported by the use of the QSR-NVivo V.12 software package (QSR International, Burlington, MA, USA). Furthermore, using the previously described analysis, an alternative presentation of the data from a cross-sectional perspective will be suggested in the Discussion section, on the basis of the categories of workers identified.

## 3. Results

### 3.1. Demographic Characteristics

The final sample consisted of 11 participants (nine paramedics and two emergency medical dispatchers), representing 37% of the entire population of trained peer helpers for this organization. Six of the 11 participants were male, with their average age being 43 years (SD = 6.1), see Table 1.

### 3.2. Qualitative Findings

**Figure 1:**
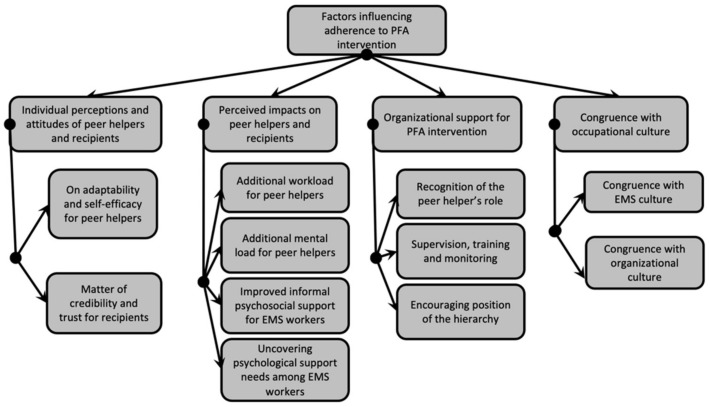
Themes and subthemes following thematic analysis.

The analysis of the interviews found four overarching themes and 11 subthemes regarding factors that may influence adherence to PFA intervention, see Figure 1. Anonymized excerpts are provided in the following sections to illustrate each theme.

### 3.3. Individual Perceptions and Attitudes of Peer Helpers and Recipients about PFA Intervention

On the basis of the participant’s responses, it was found that they held certain interpretations which oriented their actions. This finding seemed to be well illustrated by the concepts of perception and attitudes. Perception is understood in the present study as “the process by which organisms interpret and organize sensation to produce a meaningful experience of the world” [45] (p. 52) and attitude as “mindset or a tendency to act in a particular way” [45] (p. 44). Participants reported their perceptions and attitudes regarding this new PFA intervention as peer helpers. They also shared what they knew about perceptions and attitudes toward the intervention from the recipients. Those individual perceptions and attitudes helped shed light on how EMS workers perceive and act during PFA intervention. Their perceptions and attitudes also offered insight into their willingness to adhere its principles, as well as avenues for future service improvement stemming from the negative perceptions that were expressed.

#### 3.3.1. On Adaptability and Sense of Self-Efficacy for Peer Helpers

Regarding peer helpers’ perceptions and attitudes, all participants described some positive perceptions toward PFA intervention. Some mentioned how simple and easy it was to provide PFA interventions. Many perceived PFA interventions to be well suited to EMS workers’ needs.

*“It was responding to actual needs, so it’s well adapted. I think the tools that have been put in place are targeted, they are simple and because it’s simple, effective, and allows me to go straight to where I need to go, which is to respond to a need […]. Peer helpers have proven that they are a good influence for not doing well.”*

(Participant 5)

Some negative perceptions were also mentioned by a few participants, suggesting areas of improvement. Specifically, some peer helpers shared concerns regarding their own ability to adequately provide PFA intervention or concerns about how they would be accepted as peer helpers by co-workers.

*“At the beginning, it’s pretty worrisome as a program because you don’t know what you’re getting into […]. Actually, I had my doubts before applying it. I questioned its applicability; I doubted the responsiveness. I was afraid that the reactions would not be in line with what we’d learned. To get bogged down in answers or a slippery slope.”*

(Participant 3)

Concerning the attitudes stated by peer helpers, they favored adherence to the PFA intervention. They reported their investment in their role as peer helpers and their feeling of being confident when delivering PFA. Most of them were mindful to follow the directives of PFA intervention, and some described their flexibility and ability to take ownership of the intervention, enhancing their sense of self-efficacy.

*“Sometimes I don’t do the actions in order; it’s more fluid in the form of conversation. Afterward, I put everything back when I complete the form. Maybe I’m a bit rebellious; I don’t do it to the letter. I have also forgotten certain things because I didn’t have the form in front of me. I contacted people afterward or when I was doing the follow-up.”*

(Participant 7)

#### 3.3.2. Matter of Credibility and Trust for Recipients

Most participants highlighted that recipients have positive perceptions toward the PFA intervention, which is a positive predictor of a good adherence to the PFA intervention. According to these participants, EMS workers seemed to give credibility and trust to the new PFA intervention and their peer helper role.

*“The credibility of the process and the program was based on accessibility, the recruitment process, and the helping relationship offered […]. The fact that there were no cases where the peer helper broke the bond of trust between the recipient and the peer helper. These elements explain, in my opinion, why the program gained credibility.”*

(Participant 11)

The majority of participants also described some negative perceptions. According to participants, some recipients seemed to question the sustainability of PFA intervention and its credibility because of its closeness to the organization. Some also expressed concerns around potential breaches of confidentiality.

*“In the beginning, there was a lot of mistrust on the part of the paramedics because people were saying: “It’s not going to work, [Name of organization] is going to promptly drop this project, it’s not going to work.”*

(Participant 4)

Both favorable and unfavorable attitudes toward PFA interventions were identified in recipients, which have the potential to affect long term adherence. Participants predominantly perceived positive attitudes like openness, trust, and recognition of PFA interventions among recipients. Still, they reported sometimes closed attitudes (i.e., because of pride of shyness) or unwarranted personal abuse of PFA interventions (e.g., work stoppage).

*“I didn’t witness it, it’s hearsay, but people were somehow arranging to take advantage of the peer helping program to get a day off, spend two, three hours talking.”*

(Participant 11)

*“People’s openness to this is good. Of course, I didn’t make a hundred interventions, but people were open in the ones I did; they were understanding and communicative.”*

(Participant 7)

### 3.4. Perceived Impacts on Peer Helpers and Recipients

All participants described impact arising from PFA interventions after one year of implementation, allowing researchers to identify facilitators and barriers for adherence to PFA intervention over time. Some of these effects directly impact peer helpers, while others are more relevant for recipients.

#### 3.4.1. Additional Workload for Peer Helpers

The majority of participants mentioned the extent to which PFA intervention created an additional workload for them, as peer helpers, regularly infringing on their personal time. Unreasonable workloads may lead to some peer helpers feeling depleted, possibly affecting their involvement in providing PFA intervention in the long term.

*“Another point that I would improve, but I don’t know how … Maybe it’s the workload it gives us. I give an example: at one point, I met four paramedics at the same time. After that, contact all four people 24 to 48 h later. I know that’s part of our role; it comes with it, you can’t pass it up, but I don’t know if there’s a way maybe to lighten it up.”*

(Participant 7)

#### 3.4.2. Additional Mental Load for Peer Helpers

A few participants reported knowing peer helpers who were overly solicited for PFA interventions due to the uneven distribution of peer-support workload. In their opinion, this may have led to psychological fatigue or secondary traumatic stress reactions and therefore endangered their involvement as peer helpers over time.

*“There are peer helpers who have been overused. Was it because they were often available? Because the system was solicitating them all the time? Because people did not advertise themselves as peer helpers while some did? As a result, they were the ones who received calls for peer helpers all the time. I can say that some colleagues were overly solicited, and, in my opinion, this contributed to burnout.”*

(Participant 11)

#### 3.4.3. Improved Informal Psychosocial Support for EMS Workers

Many participants brought forward the idea that the training they received in the PFA program positively influences the way they routinely interact with their co-workers (i.e., authenticity). They also appeared to be more prevention-focused and careful about co-workers’ needs and limits when interacting with them. Therefore, the PFA intervention appeared to have improved informal psychosocial support for workers after a traumatic event, possibly encouraging future participation in mental health interventions.

*“If we see that there hasn’t been a traumatic event, but we see that there is a co-worker who is not doing well, […] we ask him if he wants to talk about it, then we can get pulled out of work if the person agrees to talk about it […]. They give us tips in training to detect people who would have problems, signs that would show a co-worker’s psychological difficulty, and try to see that. If they don’t want to talk to us, we say that we can change help out.”*

(Participant 6)

One participant even reported that the PFA training positively impacted their interactions with patients, encouraging him to maintain his participation as a peer helper.

*“It helped me in my work [as a paramedic], sometimes we tend to extrapolate a little too much, but focusing mainly on listening and a little less on dialogue (is important). Yes, it has served me well.”*

(Participant 1)

#### 3.4.4. Uncovering Psychological Support Needs among EMS Workers

Finally, some participants indicated how PFA intervention after a traumatic event brought to light workers’ demands for psychological support and behaviors to seek this support. It appears that, by reducing stigma and barriers to help, PFA intervention may favor adherence toward mental health interventions among EMS workers.

*“In my opinion, by creating this project [PFA intervention], we discovered many people who needed a lot of help. Initially, it was clear that it was for high-stress incidents, but soon we realized other needs.”*

(Participant 10)

### 3.5. Organizational Support for PFA Intervention

Participants highlighted several elements falling under the heading of organizational support toward PFA interventions. It appeared essential to participants that the organization demonstrates endorsement of, support for, and commitment toward the PFA program and its peer helpers to ensure long-term success.

#### 3.5.1. Recognition of the Peer Helper’s Role

A few participants named the importance of symbolic or financial recognition for their role as peer helpers. They would like to see the volunteer work they have accepted as a peer helper being recognized and valued by the organization in order to maintain their commitment over time.

*“Peer recognition is rewarding. Though, I wonder if recognition by the organization for either an improvement or an identification more. You know there are titles for everything at [Name of the organization]. I wonder if that could be something interesting or something to consider.”*

(Participant 5)

#### 3.5.2. Supervision, Training, and Monitoring about PFA Intervention

Most participants valued the clinical support they receive from the organization’s psychologist and colleagues when they have questions about their intervention with a recipient. However, clinical support is not sufficient for some of them and is not easily accessible for every peer helper. Some participants call for more regular supervision to facilitate the pursuit of their practice as PFA peer helpers.

*“I am lucky to meet regularly [the psychologist] and exchange quickly on questions or issues, but, on the other hand, I don’t think that everyone has this chance. I wonder about more meetings, closer follow-ups with her, in a group, for example […]. I think that latitude is peer helpers’ greatest strength and weakness, but you’re also left on your own, and that creates a certain emptiness.”*

(Participant 5)

The majority of participants expressed their willingness to maintain or even develop their skills as PFA peer helpers. Indeed, they suggested that the organization provide them with continuing training for PFA core actions and additional training to ensure that the quality of their interventions with co-workers is high. From a monitoring perspective, several participants reported the need for more feedback from the program, such as the number of interventions per year, impacts of their interventions, adjustments to be made to help them maintain their motivation as peer helpers.

*“It would be nice to make “wrap-ups” of the year, quarterly “wrap-ups.” To say: What worked well? What didn’t work well? What should be adjusted? Whether it’s every six months, or whether we meet once a year and say: “This year, there were that many interventions…”, “there was that much business…”. We had periods of overdraft; what could we do to cover them? I would have liked us to look back at real cases, find out how others did it and what the strengths and weaknesses were so that we could refine our interventions.”*

(Participant 10)

#### 3.5.3. Encouraging Position of the Hierarchy

Most of the participants highlighted that the organization’s hierarchy (from managers in the field to more senior managers) is currently supportive of this new practice. Massive efforts appeared to be made in terms of technicality (i.e., road clearance, dedicated room) to ensure that PFA intervention is and remains a priority and an essential component of the organizational services offered to EMS workers. This favorable positioning of the hierarchy is perceived to facilitate their work as PFA peer helpers.

*“Most of the time, on the job, we are free to do the interview and the report without really any problem, it’s unbelievable! Never a problem. I have even been removed from Priority 1 [First priority call] on occasion to be a peer helper. It’s really at the top of the ladder; I felt like it was at the top of the assignment ladder … the leaders never have a problem offering us a room or an environment.”*

(Participant 3)

However, most participants also noted episodes of interference by some managers making it more difficult for them to provide PFA interventions adequately. They reported a rejection of intervention from some managers, intrusive manager behaviors in the peer helper/recipient relationship, or uncertainty about procedures changing from shift-to-shift depending on the manager. Such inequalities in treatment may lead to frustrations among peer helpers and recipients who may be less likely to adhere to PFA principles as a result.

### 3.6. Congruence with Occupational Culture

Adherence to PFA intervention was also influenced by the level of congruence that the intervention holds with professional culture (e.g., EMS) and the specific organizational culture.

#### 3.6.1. Congruence with EMS Culture

The vast majority of participants described how they perceived PFA intervention to be compatible and conforming to EMS culture. For some, PFA intervention falls in line with their professional culture. Participants reported that PFA intervention is quick and easy to provide as it can be performed anywhere, facilitating participation for these workers. Moreover, PFA core actions are described as being relevant for EMS workers’ characteristics.

*“We are not people who are strangers to the intervention, so for us, it was easily assimilated, I think. When you have a certain number of years of experience, putting words and gestures on intervention is much easier than “Mr. and Mrs. Everybody” […]. We do a lot of psychological interventions within the population, so offering psychological interventions with co-workers is still part of the subject.”*

(Participant 10)

On the other hand, for some of the participants, PFA intervention may have led to role conflict between EMS workers and peer helper positions. Core actions of a peer helper are sometimes incompatible with professional obligations of EMS workers, particularly regarding detection and reaction to physical symptoms (i.e., hyperventilation, hypertension) or the report of suicidal thoughts or high-risk behaviors. This discrepancy with their EMS-worker role and responsibilities may lead to cognitive dissonance or a loyalty conflict, which can, in turn, be detrimental to their adherence to the PFA intervention process over time.

*“In theory, if I do an intervention, it remains confidential at the peer helper level. However, I still keep my paramedic hat on if there are clinical aspects, such as chest pain. I have to assess the chest pain. When I finish the procedure, I still have to fill out a report that the person has refused transport.”*

(Participant 9)

#### 3.6.2. Congruence with Organizational Culture

Beyond the EMS culture, several participants indicated that adherence to PFA intervention is made difficult by the specificity of their relation to the organizational structure and past organizational responses to the psychological distress of EMS workers. Participants mentioned the extent to which some members of the EMS workers reject PFA intervention due to bitterness or lack of trust toward the organization that offers it due to past organizational response.

## 4. Discussion

Using a qualitative inductive approach, the present findings reveal factors that may foster or hinder adherence regarding participation in PFA intervention among EMS workers, according to peer helpers’ perspectives. Researchers identified four themes and 11 subthemes influencing adherence to PFA intervention: (1) individual perceptions and attitudes of peer helpers and recipients about PFA intervention; (2) perceived impacts on peer helpers and recipients; (3) organizational support for PFA intervention; and (4) congruence with occupational culture.

**Figure 2:**
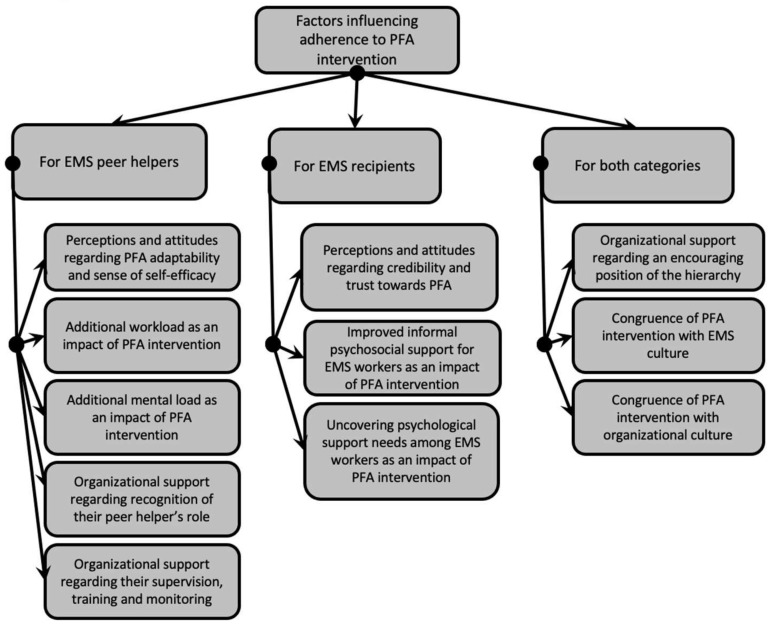
Themes and subthemes graded by category of workers.

With a cross-sectional lens, it appears that some factors can influence the adherence of both peer helpers and recipients, such as the congruence of PFA intervention with EMS culture and organizational culture as well as the approval of higher management. Other factors appear more specifically related to peer helpers’ adherence, such as perceptions and attitudes regarding the adaptability of the intervention and their sense of self-efficacy, the additional workload and mental load for peer helpers, and the recognition of the peer helper role. The need for more supervision, training, and monitoring is also likely to influence adherence of peer helpers. Finally, it appears that some factors can influence recipients’ adherence, such as perceptions and attitudes regarding the matter of credibility and trust and the perceived impacts concerning improved informal psychosocial support and the unveil psychological support needs of EMS workers. Adherence of both peer helpers and recipients is essential for PFA intervention to exist and be sustained over time in an organizational context, so it may be useful to categorize the identified subthemes in accordance with these two categories of workers; see Figure 2. Specific guidelines for each category could then emerge.

Participants identified factors that influence their adherence to PFA intervention and that help maintains their adherence over time. The question of adherence is, therefore, in the middle ground between the concepts of implementation and sustainability [33,46]. Indeed, the factors inductively gathered in the present study are consistent with the literature in both directions.

Present results regarding individual perceptions and attitudes of peer helpers and recipients suggested elements that could influence adherence to the intervention over time. For peer helpers, perceptions and attitudes regarding adaptability and self-efficacy, such as confidence to provide, the feeling of being sufficiently equipped, and providing an adapted intervention, seems to be crucial elements to maintain their participation as peer helpers over time. These are the most studied elements regarding the delivery of evidence-based psychosocial interventions [47], especially in PFA intervention [28,29,30,31]. This suggests they are the first and most essential factors organizations should consider regarding adherence to PFA intervention. For recipients, concerns about credibility and trust toward this intervention may lead to rejection or abuse and thus affect their adherence over time. In accordance with the present study, Forbes stated that PFA providers should feel confident in applying PFA, PFA core actions should be well implemented, confidentiality should be ascertained, and recipients should feel positively supported by PFA intervention [27]. Those results are also congruent with a recent systematic review of influences on implementation of peer support work for adults with mental health problems [48] as well as the first level (i.e., use of the intervention) of the Dynamic Sustainability Framework (DSF), which was developed for health interventions and implemented in organizations [46].

Further looking at the first level of the DSF [46], early perceived impacts were described on peer helpers and recipients after one year of PFA intervention. The first perceived impacts appear to be additional workload and mental load for peer helpers. Being overused and more exposed to traumatic content, by providing PFA intervention to colleagues, may lead to greater post-traumatic stress symptoms, as reported in some studies on peer support in high-risk organizations [15,49]. This may well jeopardize their adherence to FPA intervention as providers in the long run. Those results are also in accordance with Richins’s scoping review [12], which stated that early post-trauma intervention success is increased when specific need and logistical issues (i.e., workload) are identified and overcome. On the other hand, participants reported an improvement in their capacity to offer informal psychosocial support. They explained that they were more attentive to behavioral and mood changes in co-workers, more active in suggesting support, and providing a more empathetic, authentic, and listening ear. This positive impact may be linked to a generalization of the interpersonal skills learned during PFA training [28,50]. They are known to lead to a better form of support [51], facilitating recipients’ adherence to mental health intervention in the long run. As the last perceived impact, PFA intervention by peer helpers appeared to have unveiled psychological support needs among EMS workers. These last two positive perceived impacts suggest that frequent use of the intervention over time (e.g., adherence to PFA intervention), using a peer helper model, may be a good way to reduce stigma and barriers to mental health care in first responder organizations [12,15].

In findings related to overall organizational support for PFA intervention, participants described three main aspects of organizational support: recognition of the peer helper’s role, supervision, training, and monitoring of PFA intervention, and favorable position of the hierarchy. In Richins’ review, a synthesis of study outcomes found that early post-trauma interventions help emergency responders manage post-traumatic events when they are delivered in a manner supported by organizations [12]. These empirically induced results also echoed the model proposed by Forbes. Forbes and colleagues [27] suggested that organization policy supporting PFA interventions, organizational procedures, regular supervision, training follow-ups, and monitoring providers’ activity ensures a successful implementation. The theme is also congruent with the second level of the DSF (Practice setting). This level focuses on setting characteristics such as policies and procedures, human and capital resources, providing training and supervision [46]. Finally, peer-support literature also underlies that training and detailed role definition favor successful program implementation [48].

With respect to the last theme related to congruence with occupational culture participants, it was indicated that PFA intervention appears compatible with EMS occupational culture, because of its quick and flexible nature. However, they reported some tension with EMS workers’ usual role and responsibilities (i.e., reaction to physical symptoms) that need to be solved to ensure adherence over time. They also mentioned that specific organizational culture elements (i.e., lack of trust due to past organizational response toward mental health) are helpful in ensuring adherence to a new and disruptive intervention. Addressing distinctive organizational culture is also described by Richins and colleagues [12] as an important factor in order to make early post-trauma intervention models successful in emergency and other high-risk organizations. Likewise, organizational culture was identified by Ibrahim and colleagues [48] as an important factor of influence in peer support intervention’s implementation. Congruently, in Forbes’s implementation model, the culture and context within which PFA will be delivered are highlighted as an important factor to evaluate [27]. This theme also echoes Chambers and colleagues’ [46] ecological system level of the DSF, as this DSF level includes general policies or population characteristics that act as supplementary drivers for the successful sustainability of a mental health intervention.

With regard to limitations of the present study, we must recall that, although the objective of qualitative research is by no means the generalizability of its results [40], all participants come from a single EMS organization, which limits the widespread use of the present results. Organizational diversification would be critical to understanding the outcome as we found that organizational support and culture influence adherence to the PFA intervention. In addition, the transferability of the results needs to be supported by further studies to assess whether our themes are applicable to other first responders such as firefighters or police officers, and whether they can be applied to different types of mental stress in this population. A further limitation may be the opportunistic sampling strategy [36], which may have led to a selection bias. Indeed, volunteer selection may enhance the fact that those who choose to participate may share a characteristic that makes them different from non-participants leading to possible blind spots in the results. The self-report method, as well as the relational dynamics between the interviewer and the interviewee, may have led to social desirability effects that can affect the results. Moreover, deliberate inclusion in the sample of negative cases might have enhanced the quality of ours results leading to new perspectives on adherence to PFA in this context. Despite these limitations, the present results contribute to the literature regarding implementation and adherence to PFA intervention. Specifically, this study has good ecological validity as results came from peer helpers’ perspectives after one year of PFA implementation in an EMS setting. 

## 5. Conclusions

In conclusion, exploration of peer helpers and recipients’ adherence appear to be particularly relevant in studying PFA within an organizational context, as suggested by previous studies [26,52]. It will inform the organization on how to modify PFA implementation to allow to improve adherence and ensure sustainability in the long run. It may provide guidelines for adjustment in order to ensure that adherence is maintained over time, as the conditions of adherence must be present for the intervention to be sustainable [53]. Moreover, some of the themes are in line with Forbes’s implementation model of PFA in high-risk organization [27], and our themes appear to fit with the Dynamic Sustainability Framework [46] to reflect the experience of EMS peer helpers, when it comes to exploring the factors that influence adherence to PFA intervention over time. Therefore, this model appears relevant for use in future studies to optimize adherence to PFA intervention.

These study findings suggested that it is possible to act on various factors to improve peer helpers and recipients adherence to PFA implementation and ensure sustainability in EMS organizations. First, individual perceptions and attitudes of peer helpers and recipients about the PFA intervention could be worked upon by focusing on confidence for peer helpers, credibility, confidentiality, and feeling supported for recipients. Second, perceived impacts of PFA intervention should be monitored for additional workload, additional mental load, and improvement in informal psychosocial support for peer helpers, and unveiled psychological support needs among recipients. Third, organizational support should provide role recognition, supervision, training, and monitoring for PFA intervention and favorable and sustained positioning of the hierarchy. Fourth, congruence with occupational culture should be considered and strengthened. These findings will help inform both program and research design for evaluating such a program for other first responder organizations worldwide. They suggest that PFA intervention’s implementation should be flexible, tailored to the setting-specific needs, and refined if needed to favor adherence over time.

## Figures and Tables

**Table 1:** Participant demographic and professional information.

	Mean	SD
Age	43	6.1
	N	%
Sex		
Men	6	55
Women	5	45
Marital status		
Single	2	18
Married/living with partner	8	73
Divorced/widowed	1	9
Highest Educational level		
Secondary school degree	2	18
High school degree	4	36
University undergraduate degree	3	27
Graduate degree	2	18
Professional status		
Paramedic	9	82
Emergency Medical Dispatcher	2	18
Professional experience		
10–15 years	6	55
16–25 years	4	36
>25 years	1	9
Number of PFA intervention		
3–7	4	36
8–15	5	45
16–20	2	18

Note: N = 11.

## Data Availability

To protect the confidentiality of participant information, the University Institute of Mental Health of Montreal will not allow the authors to make data publicly available. Data are available upon request from Marine Tessier at Trauma Studies Center, University Institute of Mental Health of Montreal, for researchers who meet the criteria for access to confidential data.
